# AGING SERVICE AND HOSPITAL ADMINISTRATORS’ EXPERIENCE COLLABORATING ON INTERVENTION RESEARCH

**DOI:** 10.1093/geroni/igad104.1345

**Published:** 2023-12-21

**Authors:** Mary Jo McKay, Jason Wilson

**Affiliations:** Hillsborough County Aging Services, Brandon, Florida, United States; USF Health, University of South Florida, Tampa, Florida, United States

## Abstract

The next two speakers will present on the methods, benefits, and challenges of collaboration on the two RCTs described previously. One speaker is Nutrition and Wellness Manager for an aging service lead agency that oversees the county’s senior centers and nutrition sites. She and her team meet monthly with the study team for the depression trial and coordinate regularly to facilitate recruitment, intervention, outreach, and dissemination activities. One speaker is Division Chief for an academic medical center’s Division of Emergency Medicine, where he oversees collaboration on the hospital trial. Each speaker will discuss benefits of collaboration, including expanding services offered by their organization to older adults, enhancing relationships with other community organizations, and contributing to an evidence base that could impact nationwide service delivery. Each speaker will also discuss challenges that have arisen during the two trials and strategies being used to address challenges. One challenge has been recruitment. For the depression trial, the collaborators meet monthly to review recruitment progress and identify new strategies, such as a countywide phone line, connecting with other aging service organizations, and attending in-person events. For the hospital trial, research coordinators have been embedded into each hospital with primary responsibilities for using EHR and working with hospital staff to identify and enroll participants. As a result, both trials are meeting recruitment targets thus far. Other issues that have benefited from close collaboration involve COVID-19 adaptations, technical support to older adults participating in the interventions virtually, and engaging older adult volunteers and peer educators.

